# Dnmt1 regulates the myogenic lineage specification of muscle stem cells

**DOI:** 10.1038/srep35355

**Published:** 2016-10-18

**Authors:** Renjing Liu, Kun-Yong Kim, Yong-Wook Jung, In-Hyun Park

**Affiliations:** 1 Department of Genetics, Yale Stem Cell Center, Yale School of Medicine, 10 Amistad, 201B, New Haven, CT, 06520; 2 Agnes Ginges Laboratory for Diseases of the Aorta, Centenary Institute, University of Sydney, Camperdown, 2042, Australia; 3 Sydney Medical School, University of Sydney, Sydney, 2006, Australia; 4 Department of Obstetrics and Gynecology, CHA Gangnam Medical Center, CHA University, Seoul, Republic of Korea

## Abstract

DNA methylation is an important epigenetic mark that regulates gene expression. Dnmt1 plays an important role in maintaining DNA methylation patterns on daughter DNA strands. Studies have shed light into the functional role of Dnmt1 regulation in the hematopoietic and epidermal systems. Here we show that Dnmt1 is required for myogenesis. Loss of Dnmt1 results in reduced expression of myogenic genes and defects in myogenic differentiation. We have utilized a conditional knockout mouse approach to examine the functional consequences of Dnmt1 depletion specifically in the developing muscle. These mice were born runted, with smaller body weights, and reduced ability to form myotubes *in vitro*. We show that expression of Id-1, a negative regulator of myogenesis, is enhanced in Dnmt1-deficient cultures, leading to enhanced transdifferentiation of myoblasts toward the osteogenic lineage. Thus, these studies demonstrate that Dnmt1 influences cellular identity and determines lineage fidelity.

Epigenetic modification of the chromatin plays crucial roles in maintaining cellular states, genomic stability, ensuring proper gene transcription and DNA repair^1^,^2^. Methylation of cytosine residues in DNA is carried out by a class of enzymes, the DNA methyltransferases (Dnmts), that tightly regulate the initiation and the maintenance of these methyl marks^3^,^4^. Dnmt3a and Dnmt3b are responsible for establishing the *de novo* patterns of methylation during embryogenesis, while Dnmt1 is responsible for the propagation of methylation patterns. Errors in this enzymatic machinery have profound effects on development and disease^5^.

All three Dnmts are required for embryonic development, with previous studies reporting *Dnmt1*^−/−^ and *Dnmt3b*^−/−^ mice to be embryonically lethal^6^,^7^. While *Dnmt3a*^−/−^ mice survive to full term, they die around 4 weeks of age^7^. Surprisingly, embryonic stem cells (ESCs) can be derived without Dnmts and maintain their stem cell characteristics^8^. Studies have also highlighted critical roles for DNA methylation in adult stem cells. Loss of Dnmt1 in neuronal progenitors leads to global genomic hypomethylation and neonatal death^9^, while *Dnmt1*^−/−^ fibroblasts undergo growth arrest and widespread apoptosis^10^. In the hematopoietic system, Dnmt3a and Dnmt3b deficiency leads to defects in self-renewal of hematopoietic stem cells, but had no effect on cellular differentiation^11^. However, the loss of Dnmt1 resulted in self-renewal and differentiation abnormalities in the same cell type^12^. Studies in the mammalian epidermis reported a premature differentiation of epidermal progenitor cells and tissue loss in the absence of Dnmt1, further emphasizing a role of DNA methylation in maintaining the undifferentiated progenitor cell state^13^.

In skeletal muscle, the importance of DNA methylation in the control of myogenesis was shown in the regulation of master myogenic transcriptional factor, *MyoD*. *MyoD* is selectively expressed in skeletal muscle cells and its expression in non-muscle cells is suppressed by DNA methylation^14^. Demethylating agents such as 5-azacytidine can induce *MyoD* transcription and lead to myogenic conversion of non-muscle cells such as in the CH310T1/2 and NIH3T3 cell lines^15^,^16^,^17^. These studies suggest that epigenetic marking through DNA methylation plays a significant role in determining cell fate during muscle development. Limited data exists on the function of Dnmts during myogenesis. It has been reported that *DNMT1* mRNA expression in human myoblasts decreased with differentiation, and this coincided with increases in myogenic differentiation gene expressions^13^. While these results are consistent with the notion that DNA methylation in self-renewal of skeletal muscle stem cells exists, more detailed analyses are needed to formally establish this.

In the present study, we have analysed the effect of Dnmt1 depletion during murine myogenesis. We demonstrate the Dnmt1 is essential for proper myogenic differentiation and cell fate transition. Absence of Dnmt1 in myoblasts lead to reduced myogenic gene expressions and defects in myoblast fusion that results in the formation of smaller myotubes. Mice with muscle-specific deletion of *Dnmt1* are runted and display smaller body weight. We show that loss of Dnmt1 results in hypomethylation of the *Id-1* promoter, a negative regulator of myogenesis, leading to increased propensity of myoblasts transdifferentiate into the osteogenic lineage. These studies will lead to a better understanding of the epigenetic mechanisms that regulate muscle development, having implications in interventions aimed at combating musculoskeletal disorders such as muscular dystrophy.

## Results

### Loss of Dnmt1 lead to the formation of immature myotubes

**Figure 1:**
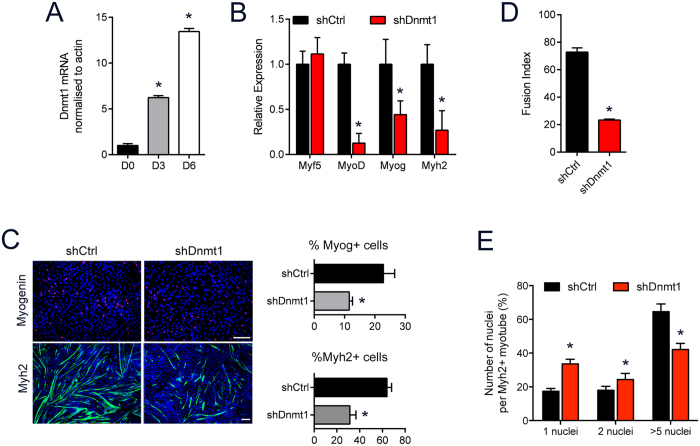
Dnmt1 knockdown adversely affects myotube formation. (**A**) Dnmt1 expression following differentiation of C2C12 cells. *P < 0.05 over D0. (**B**) qPCR analysis for myogenic genes in the shCtrl compared to shDnmt1 myoblasts. *P < 0.05 over shCtrl cultures. (**C**) Myog and Myh2 immunofluorescence in Dnmt1 knockdown upon induction of myogenic differentiation. Quantitation of the staining results showing number of Myog^+^ and Myh2^+^ cells in the knockdown cultures compared to shCtrl are shown in the bar graph. Scale bar = 50 μm. (**D**) Fusion indexes were calculated after 7 days of differentiation. *P < 0.05 over shCtrl cells. (**E**) Analysis of the number of nuclei calculated in Myh2-stained myotubes. *P < 0.05 over shCtrl myoblasts.

We first determined the expression pattern of Dnmt1 during differentiation using the well-established myogenic cell line, C2C12 (Supplementary Fig. 1). We show that *Dnmt1* transcript level was downregulated during myogenic differentiation (Fig. 1A). To study the functional significance of Dnmt1 in myogenesis, we generated a stable Dnmt1 knockdown C2C12 cell line where the myoblasts were transduced with lentiviral shRNA to target Dnmt1 and selected using puromycin (shDnmt1). Efficient reduction of Dnmt1 expression was observed in the transduced C2C12 cells (Supplementary Fig. 2A). Furthermore, knockdown of Dnmt1 was highly specific and did not affect Dnmt3a or Dnmt3b expression (Supplementary Fig. 2B). Myf5 and MyoD are required for myogenesis and are expressed in both myoblasts and myotubes. Myogenin (Myog) is an early marker of myoblasts entering the differentiation pathway^18^,^19^, and myosin heavy chain 2 (Myh2) is highly expressed when myoblasts differentiate and fuse to form myotubes^20^. We detected no difference in *Myf5* transcript levels in the Dnmt1 depleted C2C12 cultures, while *MyoD*, *Myog* and *Myh2* expression were significantly reduced (Fig. 1B). The ability of shDnmt1 myoblasts to undergo myogenic differentiation was assessed 7 days after differentiation media was added to confluent myoblast cultures. Consistent with the qPCR data, we observed decreased number of Myog- and Myh2-expressing cells in the knockdown cells which translated to significant reduction in the number of myotubes formed in the shDnmt1 cultures (Fig. 1C). We found this difference was not due to differences in cellular proliferation between the control and knockdown cultures as the number of cells were similar between groups (Supplementary Fig. 3), but rather a defect in the ability of the shDnmt1 cells to fuse (Fig. 1D,E). These data indicated that knockdown of Dnmt1 prevented differentiation of C2C12 myoblasts into myotubes.

### Dnmt1 loss coincides with a loss of myogenic gene expression in myoblasts

**Figure 2:**
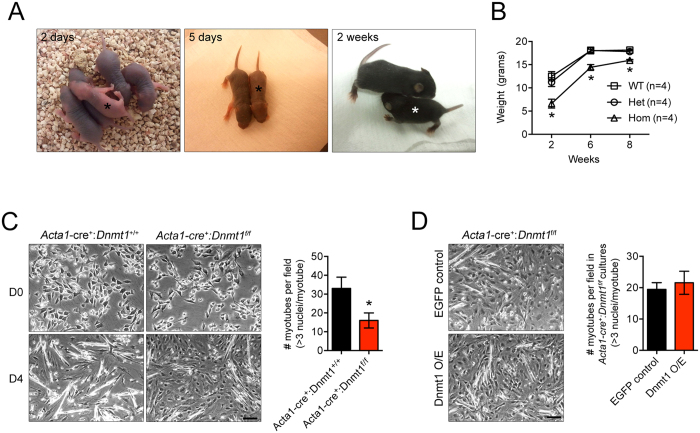
Loss of Dnmt1 results in attenuated myogenesis. (**A**) Appearance of the *Acta1*-cre^+^: *Dnmt1*^f/f^ mice and littermate controls (*Acta1*-cre^+^: *Dnmt1*^+/+^) during postnatal development. Asterisks indicate *Acta1*-cre^+^: *Dnmt1*^f/f^ mice. (**B**) Weight measurements of mice during development. *P < 0.05 over WT and Het mice of same age. (**C**) Myotube formation in the littermate control and the *Acta1*-cre^+^: *Dnmt1*^f/f^ mice. Number of myotubes per field were counted and graphed on the right. Five individual fields over three independent cultures were analysed. *P < 0.05 over *Acta1*-cre^+^: *Dnmt1*^+/+^ littermate controls. Scale bar = 100 μm. (**D**) *Acta1*-cre^+^: *Dnmt1*^f/f^ myoblasts were transduced with retroviruses expressing EGFP or overexpression (OE) of Dnmt1 for 48 hours, and cultured in differentiation media for 5 days. Scale bar = 100 μm. Number of myotubes per field (five individual fields over three independent experiments) counted in the EGPF and Dnmt1 O/E cultures are shown in the bar graph.

To further establish a role for Dnmt1 in myogenic differentiation, we conditionally deleted Dnmt1 expression in murine myoblasts by crossing the *Dnmt1*^fl/fl^ mice^12^ with a transgenic line expressing Cre recombinase under the regulation of the human skeletal alpha-actinin 1 promoter (*Acta1*-cre)^21^. The *Acta1* promoter is transcribed specifically in skeletal muscle at post-coitum day 9.5 (E9.5) coinciding with the earliest stages of skeletal muscle development and differentiation^22^. *Acta1*-cre^+^: *Dnmt1*^fl/fl^ mice were viable and born in Mendelian ratios, however the conditional knockout mice were born runted with smaller body weight compared to their littermate controls (Fig. 2A). This smaller body composition persisted to adulthood (Fig. 2B).

We are able to isolate myoblasts with >98% purity based on gene expression and staining for myogenic markers (Supplementary Fig. 4). We verified by qPCR that *Dnmt1* was efficiently deleted in the myoblasts isolated from the *Acta1*-cre^+^: *Dnmt1*^fl/fl^ mice, and had no effect on *Dnmt3a* and *Dnmt3b* expression (Supplementary Fig. 5). Consistent with our *Dnmt1* depleted C2C12 cells, we observed attenuated ability of myoblasts from the *Acta1*-cre^+^: *Dnmt1*^fl/fl^ mice to form myotubes *in vitro* (Fig. 2C). This coincided with reduced expression levels of myogenic genes in the knockout mice compared to littermate controls (*Acta1*-cre^+^: *Dnmt1*^+/+^) at baseline (Supplementary Fig. 5). To confirm these observations were indeed caused by an absence of *Dnmt1* in myoblasts, myoblasts isolated from the *Acta1*-cre^+^: *Dnmt1*^fl/fl^ mice were transduced with a *Dnmt1* over-expressing retrovirus, and myogenic gene expression then re-analyzed. *Dnmt1* levels following transduction were restored to levels similar to that of the littermate controls. Similarly, expression for *Myf5*, *MyoD*, *Myog* and *Myh2* were also increased in *Acta1*-cre^+^: *Dnmt1*^fl/fl^ myoblasts with *Dnmt1* overexpression (Supplementary Fig. 6). However, this restoration of Dnmt1 was not able to increase myotube formation in the *Acta1*-cre^+^: *Dnmt1*^fl/fl^ myoblasts (Fig. 2D). Together, these data confirm an important role of Dnmt1 in regulating myogenesis.

### Loss of Dnmt1 leads to increased activation of Inhibitor of DNA binding

**Figure 3:**
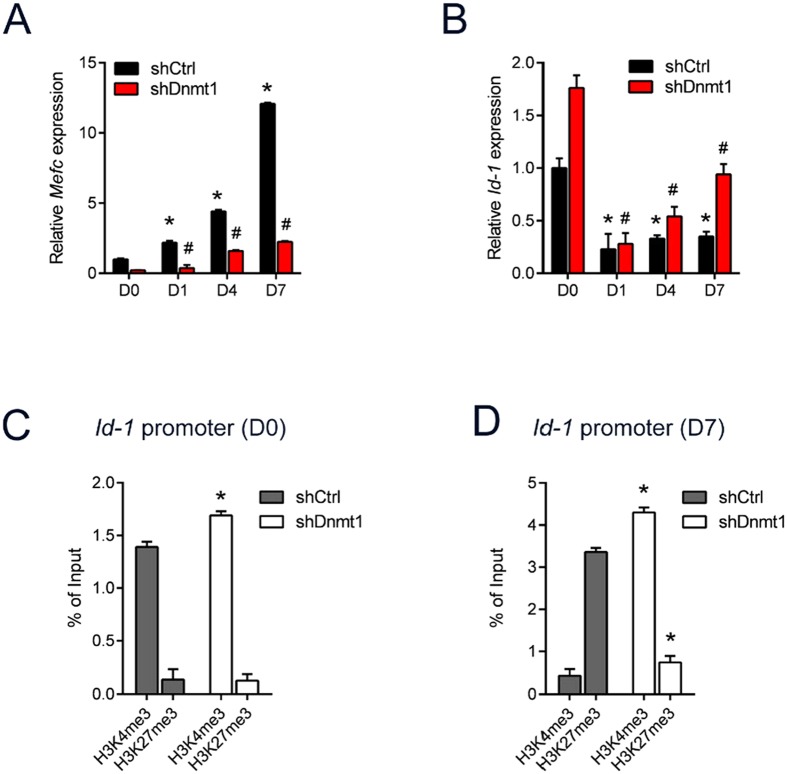
Absence of Dnmt1 leads to hypomethylation of the *Id-1* promoter. (**A**) qPCR analysis of *Mefc* expression in shCtrl and shDnmt1 myoblasts over 7 days of differentiation. *P < 0.05 over D0 shCtrl; ^#^*P* < 0.05 over D0 shDnmt1. (**B**) qPCR analysis of *Id-1* expression in shCtrl and shDnmt1 myoblasts over 7 days of differentiation. *P < 0.05 over D0 shCtrl; ^#^*P* < 0.05 over D0 shDnmt1. (**C,D**) ChIP-qPCR was performed with H3K4me3 or H3K27me3 antibodies on chromatin obtained from day 0 myoblasts (**C**) or day 7 myotubes (**D**). The precipitated DNA was amplified by qPCR using specific primers targeting a region of *Id-1* promoter ^*^P < 0.05 over shCtrl.

In addition to the basic helix-loop-helix (bHLH) myogenic factors (*MyoD*, *Myf5* and *Myog*) that are known to have critical roles in orchestrating the muscle phenotype, the *myocyte enhancer factor 2* (*Mef2*) family of genes are also required for the regulation of myogenic gene expression^23^. In line with the function of *Mef2c* during muscle maturation, we show that *Mef2c* expression is markedly increased with C2C12 differentiation (Fig. 3A). However, *Mef2c* expression in Dnmt1 depleted cells was almost 4-fold lower compared to scrambled control at baseline, and remained low throughout the duration of the differentiation (more than 7-fold lower than shCtrl cultures over the differentiation time course) (Fig. 3A). The *inhibitor of DNA binding* family of HLH proteins (*Id1-4*) are thought to affect the balance between cell growth and differentiation by negatively regulating the function of bHLH transcription factors^24^. *Id-1* is a negative regulator of *MyoD*^25^, and its overexpression impairs the ability of myoblasts to differentiate into myotubes^24^,^26^. Similar to previous reports, *Id-1* expression decreases with myogenic differentiation (Fig. 3B). However, we observed elevated *Id-1* baseline expression in the shDnmt1 myoblasts compared to shCtrl cells, as well as increasing levels of *Id-1* in the shDnmt1 cells as the cells underwent differentiation (Fig. 3B). The presence of lower expression of *Mef2c* together with higher levels of *Id-1* gene expression following differentiation could in part explain the observation of an arrest in cellular differentiation and the failure to form myotubes as seen in the *Dnmt1*-deficient cells.

Dnmt1 is known to promote DNA methylation. Therefore we sought to evaluate the chromatin status of *Id-1* loci in *Dnmt1*-deficient myoblasts. Chromatin immunoprecipitation (ChIP) assays were performed in shCtrl and shDnmt1 C2C12 cells with antibodies directed against H3K4me3 (active) and H3K27me3 (inactive) chromatin. ChIP-qPCR analysis showed similar levels of H3K4me3 and H3K27me3 between the shCtrl and shDnmt1 myoblasts at the *Id-1* promoter prior to differentiation (Fig. 3C). Following induction of myogenic differentiation, significant enrichment for the H3K27me3 mark at the *Id-1* promoter in the shCtrl cells was observed (Fig. 3D), consistent with decreased expression of *Id-1* levels in shCtrl myoblasts following differentiation as seen by qPCR. In stark contrast, the *Id-1* promoter remained in the euchromatic state in the shDnmt1 myoblasts 7 days after initiation of myogenic differentiation as demonstrated by the extensive enrichment for the H3K4me3 mark (Fig. 3D). These results are consistent with the differentiation defects observed with *Dnmt1* knockdown in C2C12 myoblasts. Therefore, these results demonstrate that Dnmt1 in myoblasts is required for methylation of *Id-1*, and that a diminished level of Dnmt1 prevents transcriptional repression of *Id-1*, leading to dysfunctional myogenesis.

### Deficiency of muscle-specific Dnmt1 leads to increased osteogenesis

**Figure 4:**
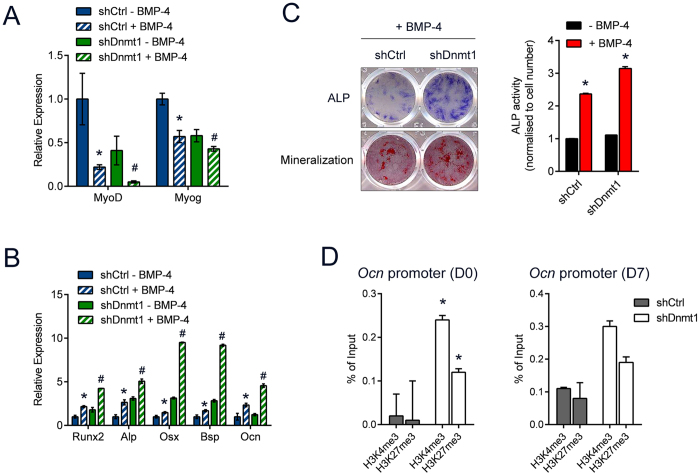
Dnmt1 knockout myoblasts undergo enhanced osteogenic differentiation. (**A**) shCtrl and shDnmt1 C2C12 myoblasts were grown in osteogenic media in the absence or presence of BMP-4 to stimulate osteogenic differentiation, and expression for myogenic markers *MyoD* and *Myog* was assessed by qPCR. *P < 0.05 over shCtrl - BMP-4; ^#^*P* < 0.05 over shDnmt1 - BMP-4. (**B**) qPCR analysis of early (*Runx2*, *ALP, Osx*) and late (*Bsp*, *Ocn*) osteogenic genes in shCtrl and shDnmt1 myoblasts without and with BMP-4. *P < 0.05 over shCtrl - BMP-4; ^#^*P* < 0.05 over shDnmt1 - BMP-4. (**C**) ALP staining performed 4 days following osteogenic differentiation, and Alizarin Red S staining performed 7 days following differentiation, in the shCtrl and shDnmt1 cells. ALP activity in shCtrl and shDnmt1 cells after 4 days of BMP-4 treatment is shown in the bar graph. *P < 0.05 over shCtrl - BMP-4. (**E**) ChIP-qPCR was performed with H3K4me3 or H3K27me3 antibodies on chromatin obtained from day 0 myoblasts or day 7 myotubes. The precipitated DNA was amplified by qPCR using specific primers targeting a region in the *Ocn* promoter. ^*^P < 0.05 over shCtrl.

Muscle cells have the potential to transdifferentiate into the osteogenic lineage in the presence of bone morphogenetic proteins (BMPs)^27^. BMP induce the expression of *Id-1*, which negatively regulates myogenesis^25^. To test whether the increased expression of *Id-1* as a result of Dnmt1 deficiency can affect the ability of myoblasts to undergo osteogenic differentiation, shCtrl and shDnmt1 myoblasts were cultured in media in the absence or presence of BMP-4. In agreement with previously published reports, addition of BMP-4 decreased the expression of myogenic genes in shCtrl myoblasts, and this reduction was further exacerbated in shDnmt1 myoblasts in the presence of BMP-4 (Fig. 4A). Concomitant with this decrease in myogenic genes was an increase in expression of early (*Runx2*, *alkaline phosphatase*, *Alp,* and *osterix, Osx*) and late (*bone sialoprotein*, *Bsp*; and *osteocalcin*; *Ocn*) osteogenic markers (Fig. 4B). Induction of osteogenic gene expression was greater in the shDnmt1 cells compared to shCtrl cultures as demonstrated by ALP staining and activity, and the ability to form mineralized nodules as assessed by Alizarin Red S (Fig. 4C). In agreement with the osteogenic differentiation data, a more transcriptionally active *Ocn* promoter was evident in shDnmt1 cells compared to shCtrl myoblasts at both baseline and following osteogenic differentiation (Fig. 4D). Taken together, these results demonstrate that Dnmt1 safeguards myoblasts against transdifferentiation into alternative lineages such as the osteogenic lineage.

## Discussion

It was hypothesised more than 30 years ago in two independent seminal papers by Riggs^28^ and Holliday and Pugh^29^ that DNA methylation could alter gene expression by influencing the binding affinities of transcription factors or other proteins to DNA. DNA methylation has been well studied in embryos and during development, but only recently has the role of Dnmts been examined in somatic cells. Concentrating on the muscular system, we found the abrogation of Dnmt1 reduced myogenic gene expression and differentiation capacities in myogenic cells (Figs 1 and 2). Previous studies have raised the possibility that *de novo* methylation carried out by *Dnmt3a* and *Dnmt3b* may also contribute to maintenance methylation in the absence of *Dnmt1*^30^,^31^. However, we did not find any compensatory up-regulation of these *de novo* methyltransferases when Dnmt1 was depleted in our cells (Fig. 3, Supplementary Figs 1 and 2), suggesting that the observed effects were specific to the enzymatic actions of Dnmt1. Interestingly, when we overexpressed Dnmt1 back into Dnmt1-deficient myoblasts, we were able to increase myogenic gene expression. However, we were unable to rescue their inability to form multinucleated myotubes. These data suggest that in addition to affecting myogenic gene expression, some other critical factors during myogenesis are controlled by Dnmt1.

During development, mesenchymal cells can either undergo myogenic, adipogenic, osteogenic or chondrogenic differentiation. Stable modifications made to the methylation pattern of DNA activate lineage-specific genes and prevent the transcription of genes from other lineages. Treatment of mouse C3H10T1/2 fibroblasts with 5-azacytidine^32^, or the overexpression of antisense RNA against *Dnmt1*^33^ induced a myogenic program in this cell type that do not normally undergo myogenesis. This effect on lineage specificity associated with the absence of Dnmt1 is consistent with our data where Dnmt1 deficiency in C2C12 myoblasts alters their cellular identity and led to enhanced differentiation into the osteogenic lineage (Fig. 4). To confirm an effect of Dnmt1 in regulating cell fate, the *Id* genes were examined for their role in transdifferentiation^34^,^35^. We further show that the *Id-1* promoter is hypomethylated in Dnmt1 knockdown cells.

Errors in DNA methylation has been linked to a number of human diseases^1^. Aberrant methylations of tumor suppressor genes or oncogenes are frequently linked to the metastatic potential of many tumour types. Mutations in DNMT3B have been linked to human ICF syndrome^36^,^37^, and abnormalities in genomic methylation patterns of DNMT3L has been linked to infertility^38^. Mutations in DNMT1 have also recently been implicated in neurodegenerative diseases^39^, while hypomethylation of the A161 allele is associated with the pathogenesis of facioscapulohumeral muscular dystrophy^40^,^41^. We have shown here that Dnmt1 plays a functional role in myogenesis, and that the *Acta1-cre*^*+*^*: Dnmt1*^*fl/fl*^ mice display a runted, dystrophic-like phenotype. Altogether, these results indicate that Dnmt1 is necessary to maintain the correct level of myogenic differentiation, and to prevent promiscuous transdifferentiation into alternate lineages. It provides a new direction for the study of myogenesis and will be interesting in future studies to determine whether Dnmt1 loss translates to premature aging of the tissue or muscular dystrophy.

## Materials and Methods

### Mouse lines

The *Dnmt1*^fl/fl^ mice were generously provided by Dr. Stuart Orkin (Children’s Hospital Boston, Harvard Stem Cell Institute, Boston, MA, USA)^12^ and the *Acta1*-Cre transgenic mice were purchased from the Jackson Laboratory, with the strain originally published by Miniou and colleagues^21^. All mice were maintained on a predominantly C57BL/6J background. *Acta1*-cre^+^: *Dnmt1*^fl/fl^ mice were generated by breeding mice heterozygous for the transgenes; Acta1-cre^+^: Dnmt1^+/+^ littermates were used as controls. Genotyping was performed on genomic DNA extracted from tail samples and PCR performed using previously published primers and PCR conditions. All experiments were approved by and carried out in accordance with the guideline of Yale University’s Institutional Animal Care and Use Committees.

### Cell culture

C2C12 myoblasts were cultured in DMEM supplemented with 20% FBS. Primary myoblasts were isolated from 8–12 week old mice as previously described^27^. Briefly, hindlimb muscles were enzymatically digested with 0.25% pronase at 37 °C for 1 hour and digestion terminated with the addition of 10% horse serum. Cells were cultured in DMEM containing 20% FBS and antibiotics. Myogenic differentiation was induced by culturing cells in 2% horse serum. Osteogenic differentiation was induced by culturing cells in osteogenic media^27^ and treated with 0 or 50 ng/ml BMP-4 (120-05, Peprotech). MTS assays were performed according to manufacturer’s protocol (Promega).

### Lentiviral production and shRNA knockdown

Dnmt1 shRNA (shDnmt1) and scrambled controls (shCtrl) lentiviruses were generated using standard protocols. Briefly, plasmids containing the control or Dnmt1 constructs were transfected into HEK293 cells using Fugene 6, and the viral supernatant collected and concentrated by ultracentrifugation. Cells were transduced for 48 hours and selected using puromycin (2 μg/ml). Surviving cells were then sequentially passaged to establish stable cell lines, and lines where more than 70% of Dnmt1 was knockdown was used for subsequent studies.

### Semi-quantitative and quantitative reverse transcriptase-PCR analysis

Total RNA were extracted from cells using the RNeasy mini kit (Qiagen) and quantified by Nanodrop. cDNA was prepared using the first strand cDNA synthesis kit (Invitrogen) according to the manufacturer’s instructions. Quantitative real-time PCR was performed with SYBR green PCR master mix (Biorad) using the Biorad C1000 thermal cycler. Samples were run in triplicate and normalised to β-actin. Primer sequences used are listed in Table 1.

### Histochemical staining and immunocytochemistry staining

Cellular viability was determined using the CellTitre 96 Aqueous One Solution Cell Proliferation Assay kit (Promega) according to the manufacturer’s instructions. Alkaline phosphatase activity and staining was detected as previously described^42^. Calcium deposits were assessed by Alizarin Red S staining^27^.

For immunofluorescence staining, cells were fixed with 3.7% paraformaldehyde, permeablized with 0.1% Triton X followed by washing and blocking with 10% FBS. Cells were then incubated with the following primary antibodies overnight at 4 °C: Myh2 (1:100) and Myog (1:50), all from DSHB. Samples were incubated with Alexa 488- and 555-conjugated goat anti-mouse IgG (Invitrogen, 1:250) and DAPI stained.

### Western blots and chromatin immunoprecipitation (ChIP)

Cell lysates were used for immunoblotting and ran on a 12.5% SDS-PAGE and transferred to PVDF membranes. Membranes were blocked with 5% skim milk and incubated in primary antibodies overnight. Secondary antibodies were incubated for 30 min. Primary antibodies used include β-actin (Santa Cruz, sc-1616), Dnmt1 (Abcam, #13537), Dnmt3a (Abcam, #13888), Dnmt3b (Abcam, #13604). Rabbit anti-mouse-horseradish peroxidise (Sigma, A0168) or goat anti-mouse-horseradish peroxidise (Fisher, 62–6520) secondary antibodies were used.

Chromatin immunoprecipitation (ChIP) was performed as previously described^43^. Briefly, 1 × 10^7^ cells were crosslinked and used for each immunoprecipitation. DNA was sheared to 200–750 bp by sonication. Protein G Dynabeads (Invitrogen) were used to immunoprecipitate the antibody-antigen complexes and antibodies against H3K4me3 and H3K27me3 were used. H3 and IgG were also included as positive and negative controls, respectively. Following cross-link reversal and proteinase K treatment, immunoprecipated DNA was extracted with phenol-chloroform, ethanol precipitated and eluted. Recovered DNA was purified with PCR Purification Kit (Qiagen) and analysed by quantitative PCR. Primers spanning the promoter regions of *Ocn*, and *Id1*, were used to detect amplification of input and immunoprecipitated DNA. Primer sequences are listed in Table 2. All analysis was performed relative to % input.

### Statistical analysis

Statistical analyses were performed with unpaired Student’s t test, with *P* < 0.05 considered significant.

## Additional Information

**How to cite this article**: Liu, R. *et al*. Dnmt1 regulates the myogenic lineage specification of muscle stem cells. *Sci. Rep.* **6**, 35355; doi: 10.1038/srep35355 (2016).

## Supplementary Material

Supplementary Information

## Figures and Tables

**Table 1:** Primers used for qPCR.

**Gene**	**Forward Primer**	**Reverse Primer**
*β-actin*	TGAAGTGTGACGTGGACATC	GGAGGAGCAATGATCTTGAT
*Dnmt1*	CCCGGCCATCCACCTCCTCA	ATGCGCACTGGTTCTGCGCT
*Dnmt3a*	GCTGCAGGGCAGAAGGGTGG	ATGGGTCGCTGACGGAGGCT
*Dnmt3b*	GGGCCCGGTACTTCTGGGGT	GGCAGTCCTGCAGCTCGAGC
*MyoD*	CCAGCATAGTGGAGCGCATCTCC	GGAGGCGACTCTGGTGGTGCATC
*Myf5*	CTCCGTGTCCAGCTTGGATTGCTT	CTGAAGAGCCAGCTCGGATGGC
*Myog*	ACCTTCCTGTCCACCTTCAGGGC	CTCGGGCTTCCGGGCTTAAGC
*Myh2*	AGTCGTGGAGTCCATGCAGA	CATGCGGTTGGAGTGGTTCA
*Runx2*	CGTCAGGCATGTCCCTCGGC	GGGGTAGGGTGGTGGCAGGT
*Alp*	CTGCGCCATGAGACCCACGG	AAGCAGGTGTGCCATCGGGC
*Ocn*	TGGCCCAGACCTAGCAGACACC	AACCCGGAGGACACATACCTGTGAG
*Id-1*	CCCGCTCAGCACCCTGAACG	TGGAACACATGCCGCCTCGG
*Mef2c*	CTGCCAGTGCGCTCCACCTC	GAGGGCAGATGGCGGCATGT
*Ccne1*	CCTTTCAGTCCGCTCCAGAA	GCTGACTGCTATCCTCGCTT

**Table 2:** Primers used for ChIP-qPCR.

Promoter	Forward Primer	Reverse Primer
*Ocn*	CTAATTGGGGGTCATGTGCT	CCAGCTGAGGCTGAGAGAGA
*Id-1*	CTTATAAAAGACTGGCTCCAGC	GGAGGCTGAGAACAGAAACAGAGTGTG
