# Supplementary material for: Dnmt1 regulates the myogenic lineage specification of muscle stem cells
Source: Sci Rep. 2016 Oct 18;6:35355. doi: 10.1038/srep35355 (PMC5082760; doi:10.1038/srep35355)
Supplement: Supplementary Information [file srep35355-s1.pdf]

SUPPLEMENTARY INFORMATION

Dnmt1 regulates the myogenic lineage specification of muscle stem cells

Renjing Liu[1,2,3], Kun-Yong Kim[1], Yong-Wook Jung[1,4], and In-Hyun Park[1,*]

1. Department of Genetics, Yale Stem Cell Center, Yale School of Medicine, 10 Amistad, 201B, New Haven. CT. 06520
2. Agnes Ginges Laboratory for Diseases of the Aorta, Centenary Institute, University of Sydney, Camperdown, Australia, 2042.
3. Sydney Medical School, University of Sydney, Sydney, Australia, 2006
4. Department of Obstetrics and Gynecology, CHA Gangnam Medical Center, CHA University, Seoul, Republic of Korea

* Correspondence: inhyun.park@yale.edu

SUPPLEMENTAL FIGURES

Supplementary Figure 1. Kinetics of C2C12 differentiation

Supplementary Figure 2. Dnmt1 knockdown in C2C12 myoblasts

Supplementary Figure 3. Effect of Dnmt1 knockdown on cell proliferation

Supplementary Figure 4. Characterisation of isolated primary mouse myoblasts

Supplementary Figure 5. Myogenic markers in the *Acta1-cre^{+}:Dnmt1$^{f/f}$* mice

Supplementary Figure 6. Overexpression of Dnmt1 in the *Acta1-cre^{+}:Dnmt1$^{f/f}$* myoblasts

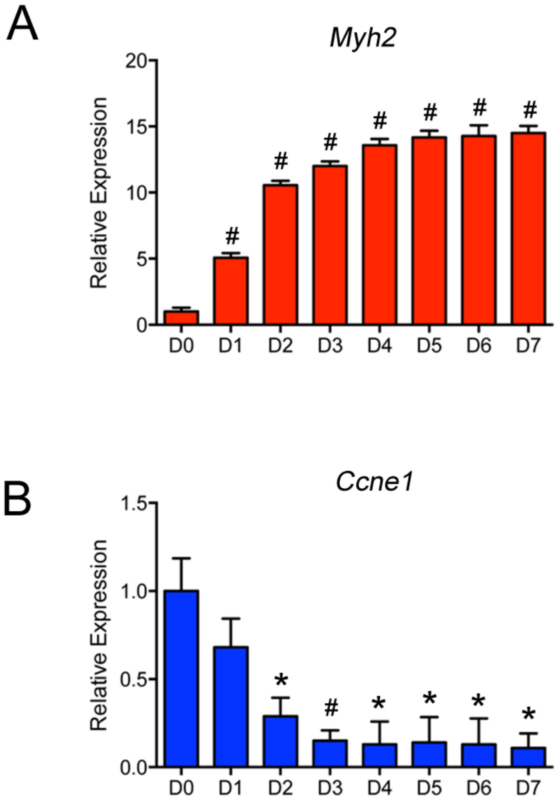

Supplementary Figure 1. Kinetics of C2C12 differentiation

Expression *Myh2* (A) and *Ccne1* (B) mRNA levels in C2C12 cells over 7 days of differentiation. **P*<0.05, #*P*<0.01 over D0 samples.

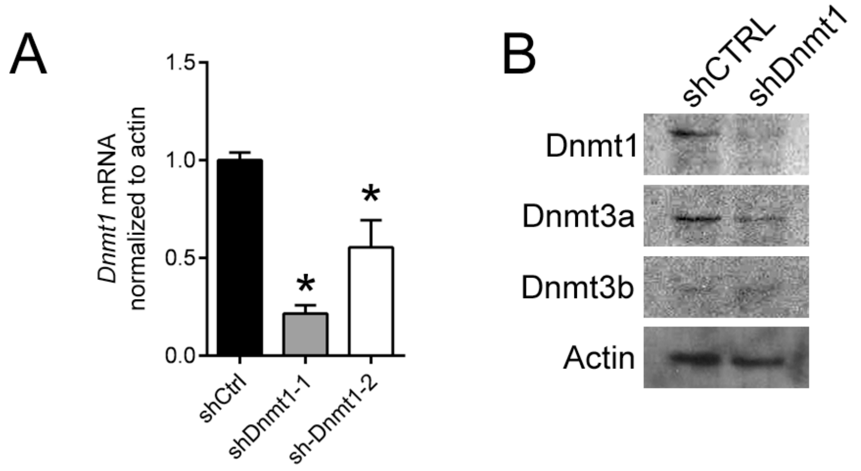

Supplementary Figure 2. Dnmt1 knockdown in C2C12 myoblasts

(A) Dnmt1 knockdown in C2C12 myoblasts using two separate shRNA constructs and qPCR was used to determine *Dnmt1* mRNA levels. **P*<0.05 over shCtrl samples. (B) Western blotting was performed to confirm knockdown and effect on Dnmt3a and Dnmt3b.

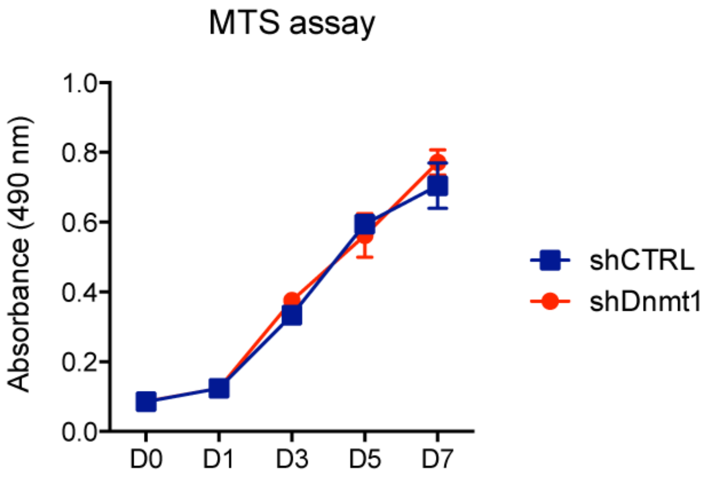

Supplementary Figure 3. Effect of Dnmt1 knockdown on cell proliferation

MTS proliferation assays were performed on shCTRL and shDnmt1 C2C12 cultures grown in growth media (D1 and D3) and differentiation media (D5 and D7).

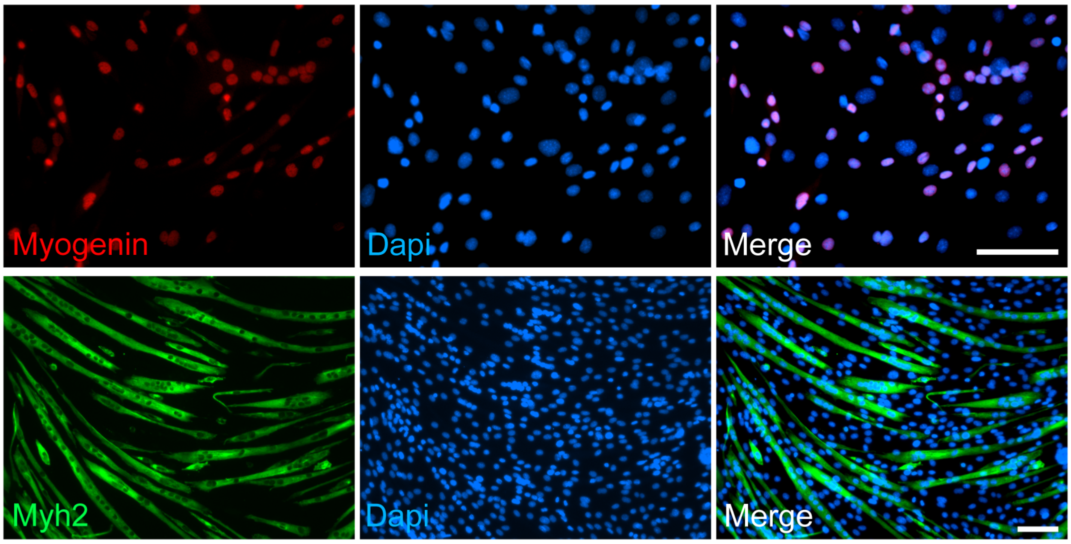

Supplementary Figure 4. Characterization of isolated primary mouse myoblasts.

Myogenin and Myh2 staining of isolated myoblasts (day 3) and differentiated myotubes (day 6) from C57BL/6 mice. Scale bar = 80 υm.

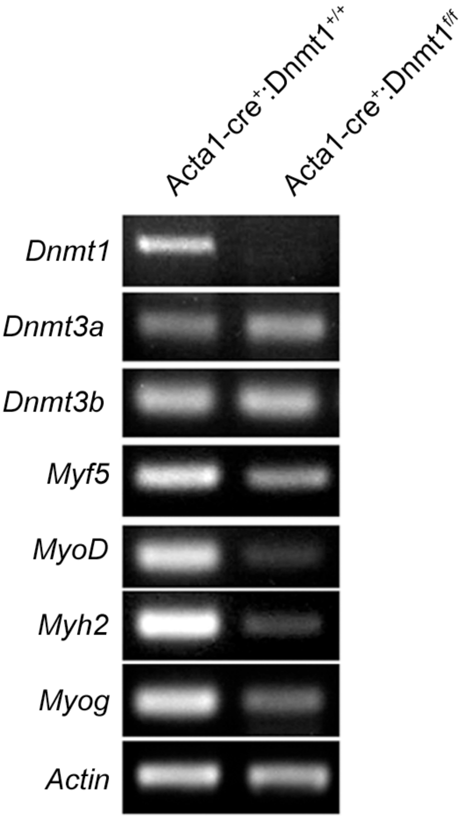

Supplementary Figure 5. Myogenic markers in the *Acta1-cre^{+}:Dnmt1$^{f/f}$* mice

RT-PCR for *Dnmt1*, *Dnmt3a*, *Dnmt3b*, and myogenic markers in the *Acta1-cre^{+}:Dnmt1$^{f/f}$* mice compared to littermate controls.

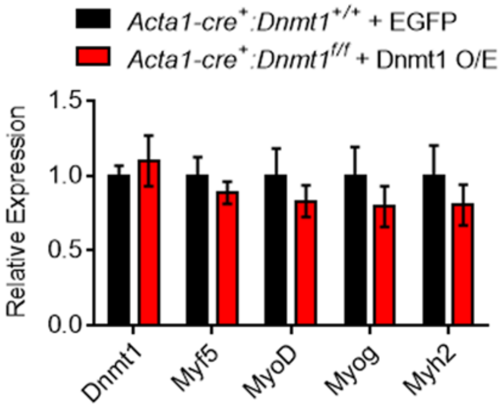

Supplementary Figure 6. Overexpression of Dnmt1 in the *Acta1-cre*$^{+}$*:Dnmt1*$^{f/f}$ myoblasts

Dnmt1 overexpressing retroviruses were transduced into myoblasts isolated from the *Acta1-cre*$^{+}$*:Dnmt1*$^{f/f}$ mice and gene expression compared to myoblasts from littermate controls transduced with retroviruses encoding EGFP. Gene expression was analysed by qPCR 72 hours post-transduction.
